# Three-dimensional cranial ultrasound and functional near-infrared spectroscopy for bedside monitoring of intraventricular hemorrhage in preterm neonates

**DOI:** 10.1038/s41598-023-30743-4

**Published:** 2023-03-06

**Authors:** Lilian M. N. Kebaya, Kevin Stubbs, Marcus Lo, Sarah Al-Saoud, Bradley Karat, Keith St Lawrence, Sandrine de Ribaupierre, Emma G. Duerden

**Affiliations:** 1 Neuroscience Program, Western University, London, ON Canada; 2 Department of Paediatrics, Division of Neonatal-Perinatal Medicine, London Health Sciences Centre, 800 Commissioner’s Road East, London, ON N6A5W9 Canada; 3 Western Institute for Neuroscience, Western University, London, ON Canada; 4 BrainsCAN, Western University, London, ON Canada; 5 School of Medicine, Queen’s University, Kingston, ON Canada; 6 Department of Medical Biophysics, Western University, London, ON Canada; 7 Imaging Program, Lawson Health Research Institute, London, ON Canada; 8 Children’s Health Research Institute, London, ON Canada; 9 Applied Psychology, Faculty of Education, Western University, London, ON Canada; 10 Department of Clinical Neurological Sciences, Schulich School of Medicine and Dentistry, Western University, London, ON Canada

**Keywords:** Neuroscience, Paediatric research, Brain imaging, Neurological disorders

## Abstract

Germinal Matrix-Intraventricular Hemorrhage (GMH-IVH) remains a significant cause of adverse neurodevelopment in preterm infants. Current management relies on 2-dimensional cranial ultrasound (2D cUS) ventricular measurements. Reliable biomarkers are needed to aid in the early detection of posthemorrhagic ventricular dilatation (PHVD) and subsequent neurodevelopment. In a prospective cohort study, we incorporated 3-dimensional (3D) cUS and functional near-infrared spectroscopy (fNIRS) to monitor neonates with GMH-IVH. Preterm neonates (≤ 32 weeks' gestation) were enrolled following a GMH-IVH diagnosis. Neonates underwent sequential measurements: 3D cUS images were manually segmented using in-house software, and the ventricle volumes (VV) were extracted. Multichannel fNIRS data were acquired using a high-density system, and spontaneous functional connectivity (sFC) was calculated. Of the 30 neonates enrolled in the study, 19 (63.3%) had grade I–II and 11 (36.7%) grade III–IV GMH-IVH; of these, 7 neonates (23%) underwent surgical interventions to divert cerebrospinal fluid (CSF). In infants with severe GMH-IVH, larger VV were significantly associated with decreased |sFC|. Our findings of increased VV and reduced sFC suggest that regional disruptions of ventricular size may impact the development of the underlying white matter. Hence, 3D cUS and fNIRS are promising bedside tools for monitoring the progression of GMH-IVH in preterm neonates.

## Introduction

Germinal matrix-intraventricular hemorrhage (GMH-IVH) is the most common neurological complication faced by preterm born neonates (≤ 32 weeks gestation), with an incidence of 25%^1–3^. Preterm birth, defined as a live birth occurring ≤ 37 completed weeks of gestation, remains the most critical risk factor for the development of GMH-IVH^4,5^. Yearly, 15 million babies are born early, accounting for 11% of births worldwide and 8% of all Canadian pregnancies^6–8^. Advancements in obstetric and neonatal care have led to improved survival of extreme preterm neonates^9–11^, who are most affected by GMH-IVH, with an incidence of 45%^12–14^. GMH-IVH is associated with a high risk of death and neurodevelopmental impairment (NDI)^14,15^.

Preterm infants with GMH-IVH face complications: posthemorrhagic ventricular dilatation (PHVD) in the short term, cerebral palsy (CP), cognitive and learning disabilities in the long term^16–19^. Preterm infants are susceptible to GMH-IVH due to an immature germinal matrix and impaired autoregulation, alongside changes in cerebral blood flow^20–24^. Moreover, the first 72 h to 1 week after birth presents a critical period of transition that ultimately predisposes these infants to complications, coinciding with the timing of GMH-IVH^15,25–27^. As a result, routine cranial two-dimensional ultrasound (2D cUS) examination is recommended for all neonates ≤ 32 weeks gestation (GA) within one week of life to identify and grade GMH-IVH; and subsequently at one month of age to identify white matter injury (WMI)^28,29^. cUS imaging is preferred due to its bedside availability, affordability and lack of ionizing radiation. cUS image quality, however, is dependent on the operator. Another cUS limitation is its inaccuracy in predicting long-term outcomes (mild GMH-IVH may lead to adverse NDI, and severe IVH may lead to good outcomes). Inconsistencies were attributed to the inability of cUS to take into account subtle WMI and the accurate site and extent of the lesion^30,31^. Compared to cUS, MRI has the advantage of providing more detail to quantify and grade IVH and other complications. However, MRI use is limited by its high cost, need for expertise in interpretation, complications associated with sedation and transport of sick neonates^32,33^. Bedside tools are needed to monitor hemorrhagic dilation of the lateral ventricles and changes in cerebral hemodynamics in the preterm neonate.

A promising bedside tool to monitor cerebral hemodynamics in preterm infants in the NICU setting is fNIRS^33^. This brain imaging technique is comparable to fMRI because it is sensitive to changes in blood oxygenation levels, an indirect marker of neuronal activity. Like fMRI, fNIRS can calculate functional connectivity, a key measure of regional brain health. Moreover, fNIRS is portable, relatively insensitive to motion and has an excellent temporal resolution.

Within 1–3 weeks of GMH-IVH, 25% of GMH-IVH cases progress to PHVD—seen on serial cUS as increasing dilatation of ventricles^34^. Untreated PHVD is harmful to the developing brain^35^. Of note, neonates with progressive PHVD, who require surgical interventions, maybe at the most risk for adverse brain and developmental outcomes^36^. The management of progressive PHVD includes temporizing measures, such as lumbar taps, ventricular taps (VT), Ommaya reservoirs or external ventricular drains (EVD), and subsequently, if necessary, a permanent ventriculoperitoneal shunt placement^37^. While PHVD treatment remains challenging in the preterm population, recent evidence supports an earlier timing for surgical intervention^38,39^.

Precise measurement of the degree of PHVD to guide timely intervention is vital. Ongoing research has shown the utility of three-dimensional cranial US (3D cUS) volumetric thresholds to guide intervention^40^. A follow-up study of infants with PHVD showed that ventricular volumes (VV) affected outcomes^41^. Findings suggest that VV may be an important biomarker for preterm infants with severe GMH-IVH^40^.

With more preterm infants surviving but with unchanging morbidity rates, this longitudinal study's ultimate goal was to identify 3D cUS and fNIRS markers that predict intervention thresholds for infants with PHVD. Hence, we used 3D cUS and fNIRS to monitor preterm infants (≤ 32 weeks GA) with GMH-IVH. The overall hypothesis was that 3D cUS ventricle volume changes would be associated with regional changes in spontaneous functional connectivity (sFC). Improved understanding of the association between VV and sFC using bedside monitoring methods may improve the prediction of intervention thresholds in neonates with GMH-IVH. We had two main aims for this study. The first aim was to assess the association of VV with sFC in preterm neonates with IVH assessed using 3D cUS and multichannel fNIRS. The second aim was to examine longitudinal assessments of 3D cUS VV in neonates with severe GMH-IVH and PHVD who underwent CSF diversion procedures and how they predict sFC.

## Methods

### Study setting and patients

This prospective cohort study was conducted at the Level 3 Neonatal Intensive Care Unit (NICU) in Ontario, Canada, from January 1, 2021, to June 30, 2022. The Health Sciences Western University’s Research and Ethics Board approved the study. The study was conducted in accordance with the Declaration of Helsinki. Families provided informed consent. Study participants were infants ≤ 32 weeks’ GA, born at or referred to London Health Sciences Centre (LHSC) NICU, and admitted with a diagnosis of GMH-IVH^42^ made by the most responsible physician on the infant’s first routine 2D cUS.

Infants (≤ 32 weeks’ GA) receive a routine cUS within 1 week of age as part of standard care; after that, a follow-up cUS at 1 month^28^. For neonates demonstrating PHVD, 2D cUS is more frequent (once a week). The decision for neurosurgical interventions is left to the discretion of the treating paediatric neurosurgeon—but is primarily based on clinical signs and symptoms and increasing ventricle volumes.

### Data collection

#### Demographic and clinical data

Maternal and neonatal data were abstracted from electronic medical records and charts. Antenatal and perinatal data: maternal age, medical conditions, place of birth, corticosteroids administration, mode of delivery, GA at birth, birth weight, sex, delayed cord clamping, invasive ventilation at birth, cord pH, Apgar score at 1 and 5 min.

Prematurity-related clinical morbidities: patent ductus arteriosus (PDA) requiring treatment, sepsis, hypotension requiring inotropes, bronchopulmonary dysplasia (the need for supplemental oxygen at 36 weeks' postmenstrual age), periventricular leukomalacia (PVL).

Ventricular measurements and fNIRS data: 2D cUS GMH-IVH staging (right, left), 3D cUS ventricle volumes (left, right and total), postnatal course during each measurement and resting state fNIRS data. For infants with PHVD, before and after each CSF diversion procedure: head circumference, weight, hemoglobin, respiratory support, tap volume, the need for VP shunt and brain MRI for those infants who went on to have one.

#### 3D cUS system and ventricle volume acquisition

**Figure 1:**
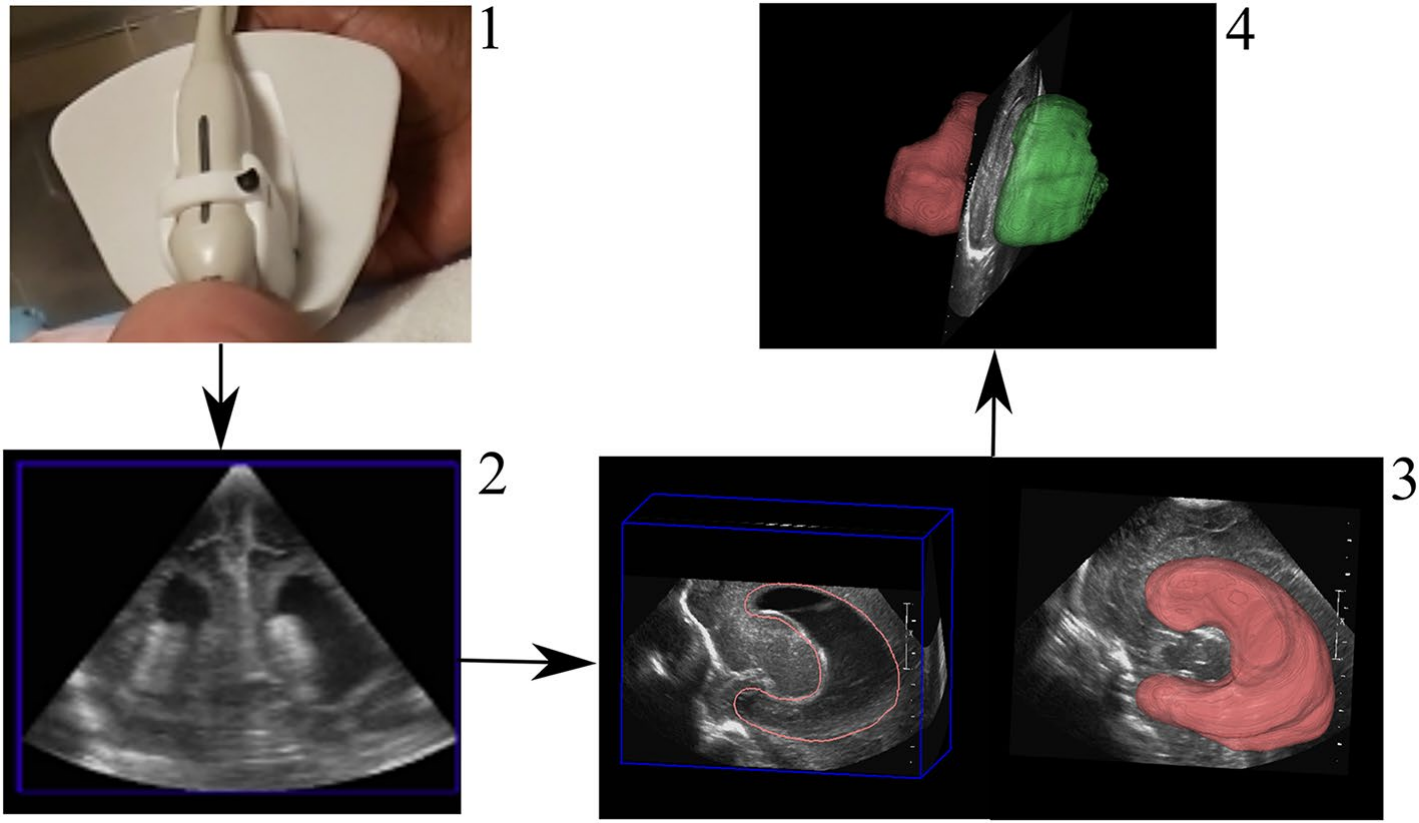
Three-dimensional cUS acquisition process: (1) Head, with US probe placed on anterior fontanelle. (2) cUS image (coronal plane) (3) lateral ventricle segmentation (sagittal plane) (4) segmented ventricles (left—green; right—red), from which a VV is provided.

We used a 3D cUS system to image the lateral ventricles attached to a clinical 2D cUS machine (HDI 5000, Philips, Bothel WA) with an appropriate conventional probe (C8-5, Philips, Bothel WA). The 3D cUS system comprises a handheld motorized device housing a 2D cUS probe (Fig. 1). The probe was placed on the infant’s anterior fontanelle (the soft part of the brain), with the infant lying supine. After initiating the scan, the device tilted the probe on an axis at the probe tip. 2D cUS images were acquired into a computer via a digital frame grabber (Epiphan DVI2USB 3.0) and reconstructed into a 3D image^40^. Total bedside scan lasted 5–10 min, accounting for infant movement and large ventricles. We manually segmented cUS images using in-house developed software. Segmentation of the lateral ventricles was performed in two planes (sagittal and coronal) by trained researchers (LMNK, ML, BK and SA) and verified by a Paediatric Neurosurgeon (SdR). The software created a 3D image of the lateral ventricles, from which a ventricle volume measurement was provided^43^. This system has been validated for geometric validity and volumetric measurements^44^. Depending on VV, the infants underwent sequential 3D cUS, weekly or biweekly, until discharge.

#### fNIRS data acquisition

**Figure 2:**
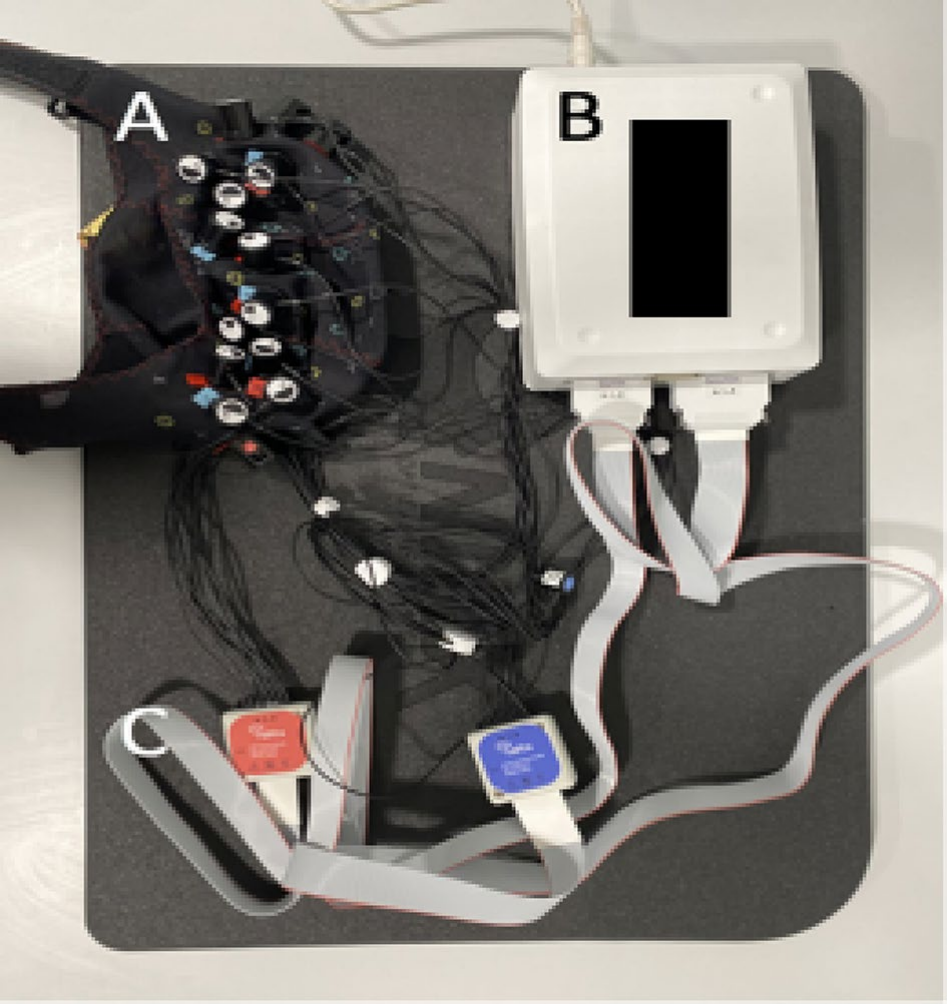
fNIRS acquisition system. (**A**) fNIRS cap. (**B**) NIRSport2 system. (**C**) Optodes.

While infants lay in the incubator, we acquired fNIRS signals using a multichannel NIRSport2 system (NIRStar Software v14.0, NIRx Medical Technologies LLC, Berlin, Germany) at a sampling rate of 10.17 Hz (Fig. 2). We inserted eight sources and eight detectors into predefined cap areas, with ten channels (22 mm separation) defined for each hemisphere (20 total). The optodes were placed over the prefrontal and sensorimotor regions. The first 16 sessions out of 182 used a slightly different montage, so all datasets have been merged to contain 20 out of 24 possible channels (Supplementary Fig. 1). Each light source contains two LEDs that emit at 760 nm and 850 nm. The fNIRS cap was positioned on the infant’s head according to the manufacturer’s guidelines. Resting-state fNIRS were acquired for a minimum of 2.5 min and a mean of 6.8 min^45^.

#### Measurements’ sequence and timing

We performed the fNIRS recording first, followed by 3D cUS at the infant’s bedside. Infants with severe GMH-IVH who demonstrated worsening PHVD, requiring a VT or EVD, underwent 3D cUS and fNIRS before and after the intervention (on the same day). Hence, all subjects had multiple 3D cUS and fNIRS measurements.

#### fNIRS patient exclusions and subgroups

Three patients with fewer than two valid sessions were excluded. The remaining patients were divided into two groups: those who did not undergo CSF diversion and those who required CSF diversion. The data from these two groups were analyzed separately because VV was reduced by the CSF diversion, which could have altered its relation to sFC.

#### fNIRS subsample selection

Each recording was reduced to the contiguous 2.5-min subsample^45^ with the highest data quality using in-house scripts and metrics adapted from QT-NIRS (formerly NIRSPlot)^46^. First, abrupt motion spikes were interpolated using an established spline-based method adapted from Homer2^47,48^. Scalp coupling index and peak spectral power were calculated in 5-s windows with 50% overlap for a cardiac range of 90–210 beats per minute. Selections were then made such that scalp coupling index and peak spectral power were maximized while segments with signal dropout (from optodes lifting off the scalp) were avoided.

#### fNIRS channel exclusions

We excluded channels within each dataset where cardiac pulsation was absent, or other issues were manually identified. Cardiac pulsation was detected using a novel method that combines the scalp coupling index with patterns in the frequency domain. Manual corrections were applied based on visual inspection. 14.8% of channels were excluded due to absent cardiac pulsation, excessive motion, and/or signal dropout. An additional 7.3% of channels were excluded due to a hardware malfunction in source 8 that impacted 3 channels in the right hemisphere. These exclusions resulted in variable channel validity across sessions and between patients. Channels that did not meet a minimum sample size threshold (No CSF Diversion: 10 out of 23 patients; CSF Diversion: 5 out of 7 patients) were indicated as having “low confidence” and were excluded from analyses.

#### fNIRS data preprocessing

Brain AnalyzIR Toolbox^49^, Homer2^47^, and in-house MATLAB scripts were used for fNIRS data processing. Following the selections outlined above, the data were converted to optical density and then to HbO and HbR using the Brain AnalyzIR Toolbox^49^. Age-appropriate partial pathlength factors (0.1063 for 760 nm and 0.0845 for 850 nm) were used in the modified Beer–Lambert Law, which yields estimates of relative concentrations of oxygenated (HbO) and deoxygenated hemoglobin (HbR)^50^. Additionally, channel lengths were scaled to the cap size used during each session. The data were then converted to total hemoglobin (HbT = HbO + HbR), which has been shown to have improved sFC reproducibility across sessions and subjects^48^. Temporal filtering and global signal regression were performed together in a nuisance regression as outlined in Blanco et al.^51^. The regression model included Fourier terms above 0.09 Hz and the first four-order Legendre polynomials, similar to a bandpass around 0.004–0.09 Hz. Additionally, the average fNIRS signal was included to regress out both motion and physiological noise. Regression was performed on HbO and HbR independently to account for physiological differences^51^. We similarly did not apply pre-whitening for reasons outlined in Blanco 2018: they could replicate prior findings without pre-whitening, observed a disruption in the antiphase relationship between HbO and HbR following pre-whitening, and lastly, results following pre-whitening were highly variable^52^. The final data were resampled to 1 Hz before calculating connectivity (see Supplementary Figs. 2 and 3).

#### Spontaneous functional connectivity

The Brain AnalyzIR Toolbox’s robust correlation method was used to calculate sFC^49^. To validate the sFC, group summary figures were generated as follows: correlations were first averaged across sessions to yield one mean value per channel pair per patient; a single-sample t-test was performed on each channel pair to evaluate sFC ≠ 0; the resulting t-map was drawn over the montage.

#### Relating spontaneous functional connectivity to ventricle volumes

Impairments were expected to manifest as increased independence in the channel signals (e.g., due to disrupted pathways), which would then yield channel-to-channel correlations closer to zero. This reduction in magnitude would be reflected as a decrease in the connectivity of correlated channel pairs and, conversely, as an increase in anti-correlated pairs. Absolute sFC (|sFC|) was used to evaluate overall disruptions in connectivity regardless of individual polarities. (i.e., convergence on zero).

The relation of |sFC| to VV was assumed to be linear and was evaluated by estimating the slope of |sFC|/VV. We did not use correlation because several patients had as few as two sessions. The |sFC|/VV slope was estimated using three incremental methods. All three methods were calculated independently for each channel pair using VV from the corresponding hemisphere’s ventricle. The first method used simple linear regression to estimate each patient’s slope. The second method used linear mixed-effects modeling (LME), which better accounted for patient variability and outliers. The third method used LME and additionally accounted for changes in both |sFC| and VV across GA.

A t-map was generated for each method evaluating |sFC|/VV ≠ 0. Positive values indicate where |sFC| increases/decreases with VV, and negative values indicate where the relation is inverted (e.g., decreasing |sFC| as VV increases). For a broad comparison, we performed a subsequent t-test (|sFC|/VV ≠ 0) on the group-level slopes from all channel pairs (independently for each hemisphere, for each method). This test indicates where significant trends were observed at the hemisphere level.

#### Longitudinal case studies in patients requiring CSF diversion procedures

For each case study, we present |sFC| and VV sessions across GA and indicate each CSF diversion procedure's times. As a middle ground between presenting every channel pair and collapsing to hemispheres, |sFC| are presented from four fully symmetrical functional clusters identified in the sFC validation t-maps. Each cluster contains four channel pairs derived from five channels (Supplementary Fig. 5).

### Statistical analysis

SPSS v.28 (IBM Corp., Armonk, NY, USA) was used for all statistical analyses pertaining to demographic data. All other analyses were performed using MATLAB R2020b (The MathWorks Inc., Natick, Massachusetts, USA).To address our first aim, we examined the association between VV and |sFC| by estimating the linear relation (slope) of |sFC| across VV at the group level. To address our second aim, we investigated |sFC| and VV across time as individual case studies only in infants who underwent CSF diversion procedures. Models were adjusted for relevant covariates and confounding variables. As we had a single hypothesis and did not make direct comparisons across the groups or methods, an alpha level s of 0.05 was considered statistically significant.

## Results

### Study population

**Figure 3:**
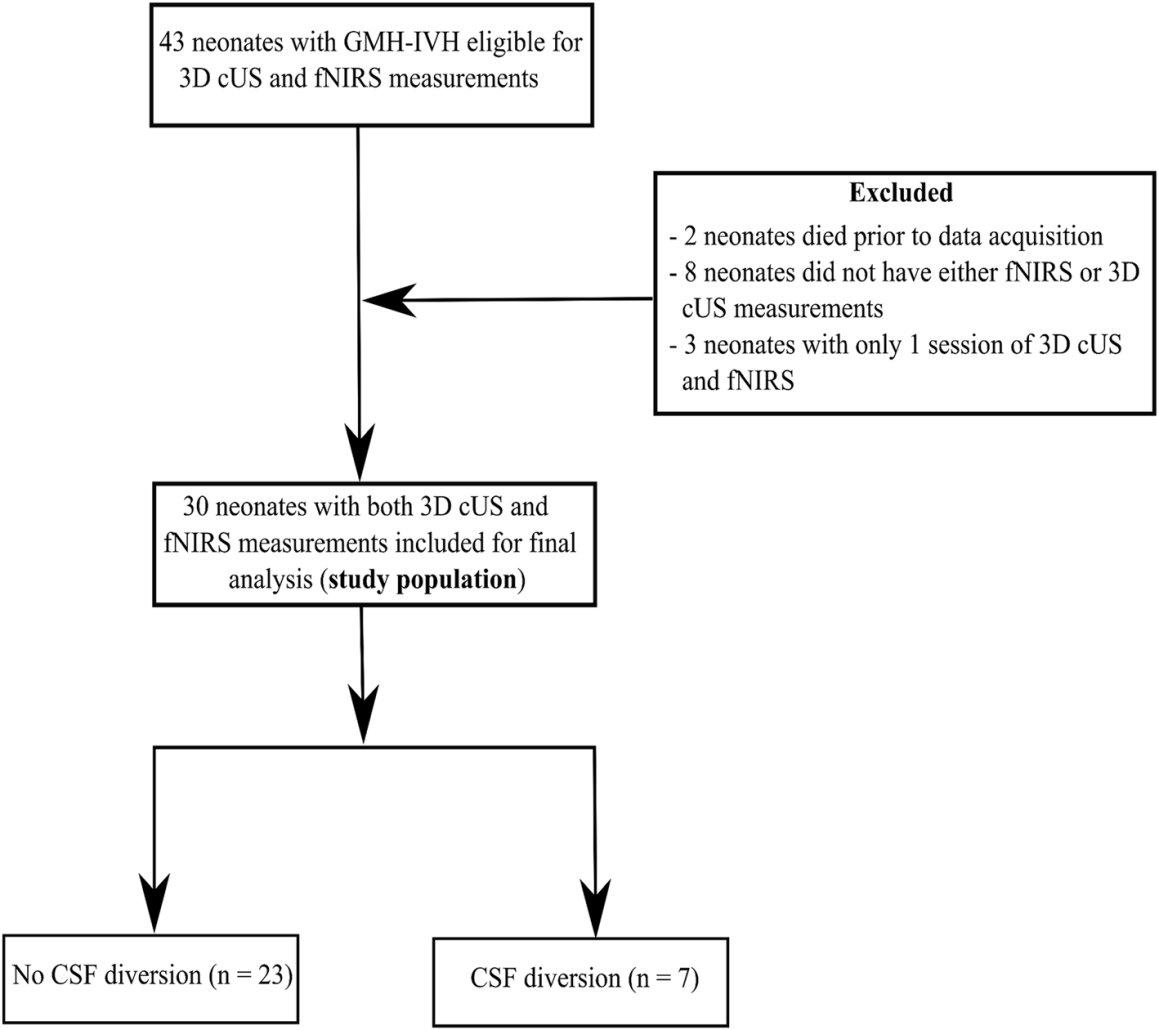
Study flow diagram.

During the study period, 43 preterm neonates (GA ≤ 32 weeks, with GMH-IVH) were eligible for 3D cUS and fNIRS monitoring. We excluded two neonates (extremely low for gestation (ELGA), < 26 weeks) following their death during the first week of life. The neonates died after a decision for palliative care in the NICU and before data acquisition. The decision to redirect care was based on the degree of prematurity, the severity of GMH-IVH and other prematurity-related complications. We excluded seven neonates because they lacked fNIRS measurements, and in another neonate, it was impossible to obtain 3D cUS measurements. In addition, three patients with fewer than two viable sessions were excluded. Thus, our study population consisted of 30 preterm infants with 3D cUS and fNIRS measurements. The study flow diagram is presented in Fig. 3. The remaining 30 neonates were divided into two groups: those who did not undergo CSF diversion (n = 23, "No CSF Diversion" group) and those who required CSF diversion (n = 7, "CSF Diversion" group).

Both groups were comparable in GA at birth (mean GA = 26.6, SD = 2.59 in no CSF diversion group versus mean GA = 26.6, SD = 3.1 in the CSF diversion group). Most infants were born at LHSC (24, 80.0%), and 16 (53.3%) were female. Twelve mothers (40.0%) received a complete course of antenatal corticosteroids, with only 1 (14.3%) in the CSF diversion group. Most mothers presented in spontaneous preterm labour and did not benefit from antenatal corticosteroid interventions. A high proportion of infants (20, 66.7%) remained intubated during the first week of life. In addition, during their NICU stay, the majority of the study participants in both groups had prematurity-related complications: hemodynamically significant patent ductus arteriosus (17, 56.7%), clinical or culture-proven sepsis (25, 83.3%), severe retinopathy of prematurity (18, 60.0%). Table 1 shows demographic characteristics and clinical data.

**Table 1:** Demographic and clinical data of the study cohort (n = 30).

Characteristics		No CSF diversion (n = 23)	CSF diversion (n = 7)	P-value	
Neonatal characteristics					
Gestational age at birth (weeks, SD)		26.61 ± 1.06, SD = 2.59	26.57 ± 2.30, SD = 3.10	0.978	
Birth weight (g)		976.1 ± 125.2, SD = 306.4	1,088.7 ± 392.2, SD = 529.43	0608	
Head circumference at birth (cm)		24.8 ± 1.09, SD = 2.7	26.9 ± 6.3, SD = 8.47	0.529	
Male sex (%)		10 (43.5)	4 (57.1)	0.109	
Apgar at 5 min		6.09 ± 0.99, SD = 2.4	7 ± 1.54, SD = 2.1	0.349	
Inborn (%)		21 (91.3)	3 (42.9)	0.0002	
Cesarean section delivery (%)		9 (39.1)	2 (28.6)	0.035	
Maternal characteristics					
Antenatal corticosteroids (complete course) %		11 (47.8)	1 (14.3)	0.004	
Maternal age (years)		31.48 ± 2.65, SD = 6.5	29.57 ± 4.9, SD = 6.6	0.519	
Neonatal complications					
Invasive ventilation > 1 week (%)		15 (65.2)	5 (71.4)		0.025
hsPDA (%)		12 (52.2)	5 (71.4)		0.090
GMH-IVH grade^42^	Grade I: 7 (30.4)		Grade I: 0 (0.0)		0.015
	Grade II: 10 (43.5)		Grade II: 2 (28.6)		
	Grade III: 4 (17.4)		Grade III: 2 (28.6)		
	Grade IV: 2 (8.7)		Grade IV: 3 (42.9)		
Culture-positive or clinical sepsis (%)		19 (82.6)	6 (85.7)		0.009
Hypotension, inotrope use (%)		6 (26.1)	4 (57.1)		0.527
BPD (%)		8 (34.8)	4 (57.1)		0.248
PVL (%)		7 (30.4)	5 (71.4)		0.564
VP shunt (%)		0 (0.0)	3 (42.9)		–
Death before discharge (%)		0 (0.0)	1 (4.3)		–
Number of sessions (Mean, median)		3.7, 3	9.6, 11		–

*RDS* Respiratory Distress Syndrome, *hsPDA* hemodynamically significant Patent Ductus Arteriosus, *PVL* periventricular leukomalacia, *VP shunt* ventriculoperitoneal shunt, *Session* time of 3DcUS and fNIRS acquisition.

Out of the 30 neonates enrolled, 19 (63.3%) had mild GMH-IVH, while 11 (36.7%) had severe GMH-IVH. Severe GMH-IVH was defined as GMH-IVH grade III or PHVI. Seven infants had CSF diversion procedures (ranging from 2 to > 15 sessions per neonate). Of these, 3 infants (42.9%) had a VP shunt inserted before discharge. Of the seven neonates in the CSF diversion group, 2 (28.6%) had grade II IVH, 2 (28.6%) had grade III IVH, and 3 (42.9%) had PHVI.

### Validation of spontaneous functional connectivity

**Figure 4:**
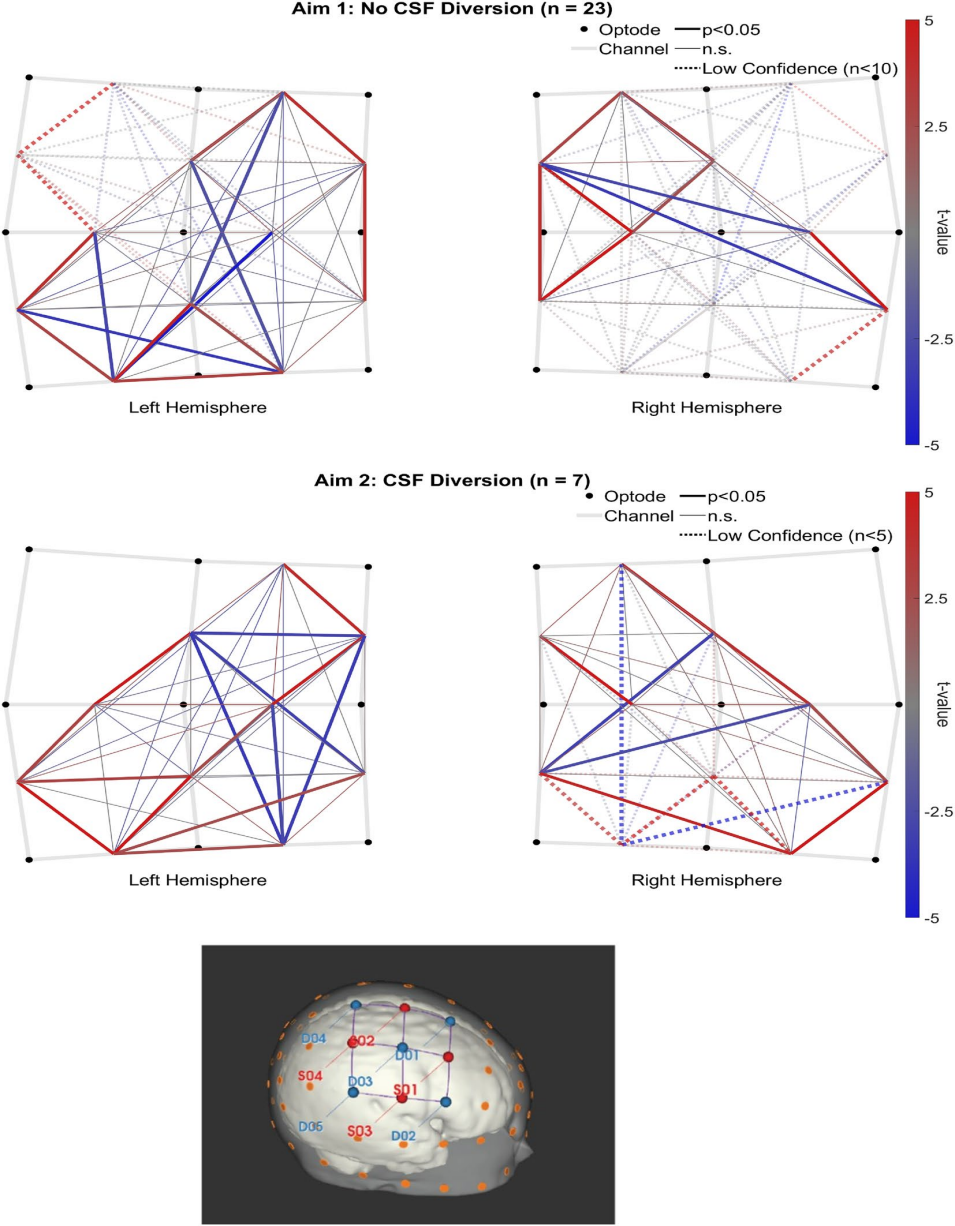
Group level sFC > 0 t-maps (top row, “No CSF Diversion group”, n = 23; bottom row, “CSF Diversion” group, n = 7). A montage of the neonatal head (right hemisphere only) is included at the bottom.

It was important first to establish the presence of sFC and identify any existing trends. Figure 4 depicts t-maps for sFC > 0 in each patient group. The 23 neonates that did not require CSF diversion had between 2 and 8 sessions (mean 3.65, SD 1.82), and the 7 who needed CSF diversion had between 3 and 15 sessions (mean 9.57, SD 4.32). In both groups, the t-maps include clusters of adjacent channels that were positively correlated, anti-correlations between those clusters, and many significant anti-correlations between hemispheres (see Supplementary Fig. 4 for anti-correlations between hemispheres). The sFC patterns in HbO and HbR closely matched the presented HbT results (see Supplementary Figs. 7 and 8).

### Ventricle volumes and spontaneous functional connectivity

**Figure 5:**
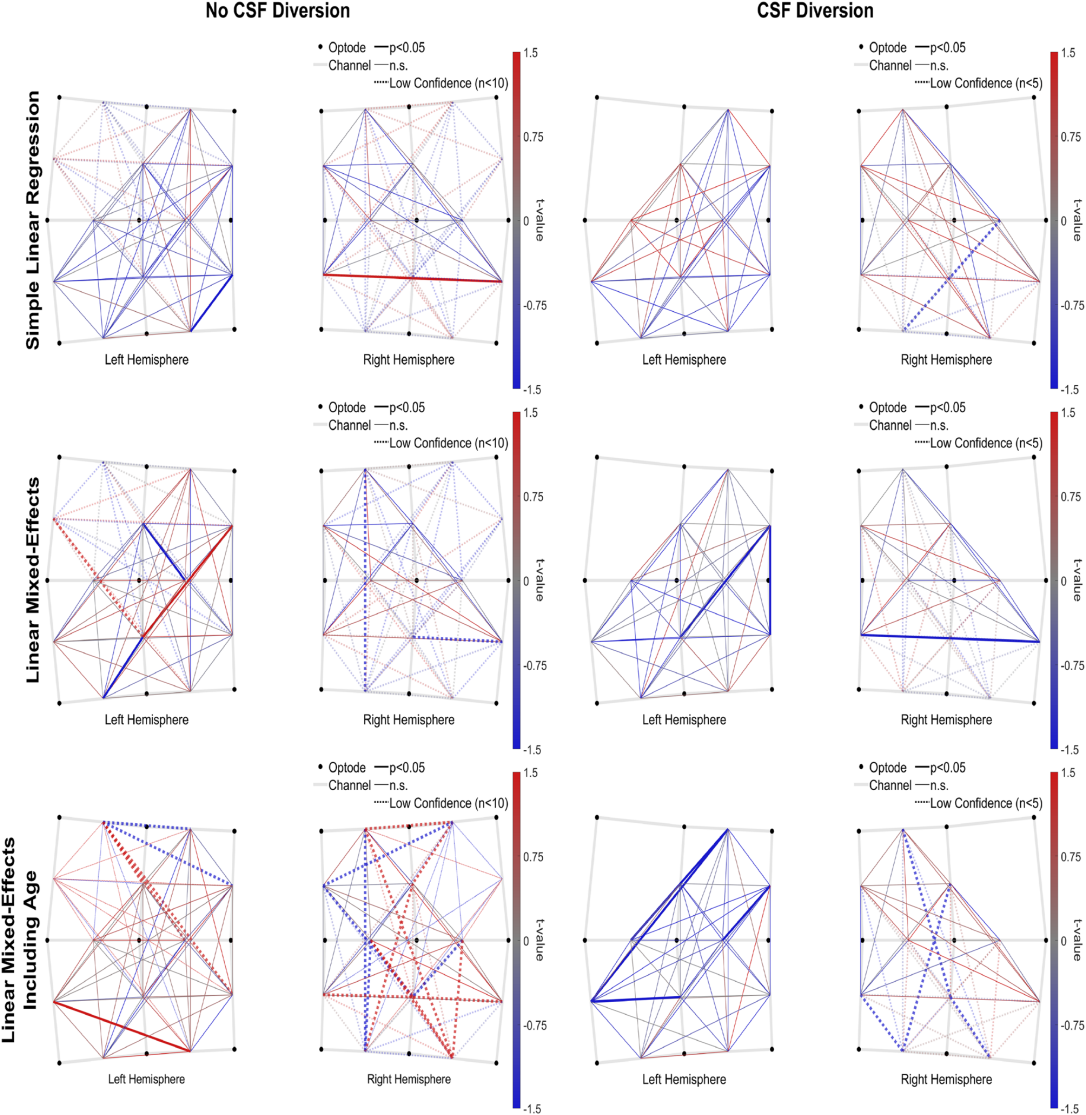
Group level |sFC|/VV t-maps from each slope method (left = “No CSF diversion” group, n = 23; right = “CSF diversion” group, n = 7); Rows: top = simple linear regression, middle = LME, bottom = LME + GA.

**Figure 6:**
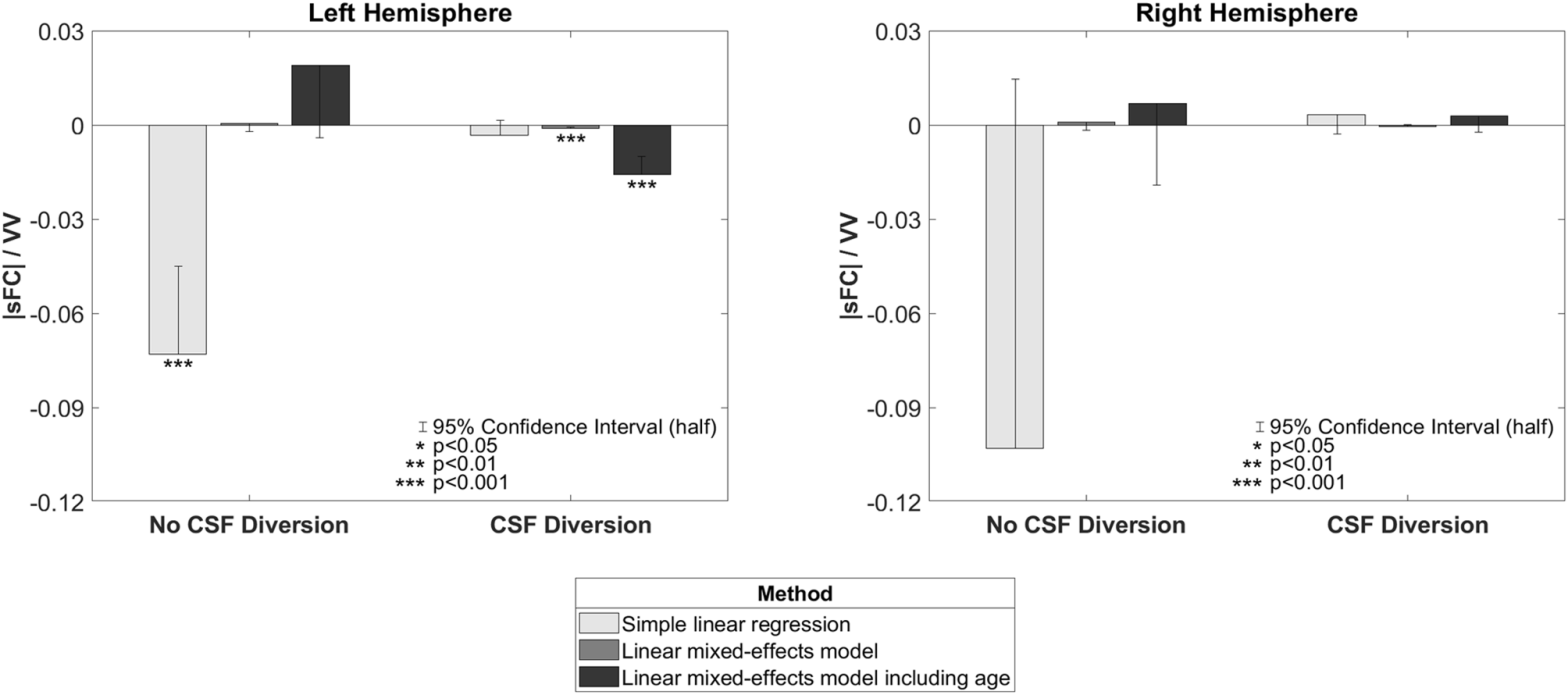
Hemispheric trends in group level |sFC|/VV from each slope method.

Our first aim, the association of VV with sFC in preterm neonates, was examined using 3D cUS and multichannel fNIRS. In the “No CSF Diversion” group, the linear regression method produced a widespread negative |sFC|/VV (see Fig. 5) that was significantly less than zero in the left hemisphere (slope = − 0.0729, p < 0.001) and trending similarly in the right (slope = − 0.1030, n.s.). No significant slopes were observed when using either LME method with this group, but a positive trend was observed across the three methods. These results are outlined in Figs. 5 and 6.

In the “CSF Diversion” group, |sFC|/VV trended negatively in the left hemisphere across linear regression (slope = − 0.0032, n.s.), LME (slope = − 0.0012, p < 0.001), and LME additionally accounting for GA (slope = − 0.0159, p < 0.001). Several channel pairs (mostly adjacent) were independently significant in both LME results. However, the right hemisphere was inconsistent and non-significant across the three methods.

To provide context to the observed trends in |sFC|/VV, we performed a post-hoc investigation of possible trends in VV and |sFC| across GA. In each group, a pair of linear mixed-effects models were used to evaluate VV and |sFC| independently. No significant linear effects of GA on VV or |sFC| were observed when accounting for patient variability in this model.

For completeness, we repeated this analysis on both HbO and HbR. The pattern of results in both closely matched the presented HbT results (i.e., the slope of |sFC|/VV obtained from LME + GA was positive in the “No CSF Diversion” group and negative in the “CSF Diversion” group). These results have been added as Supplementary Fig. 9.

### Longitudinal assessments of 3D cUS ventricular volumes and sFC prediction

**Figure 7:**
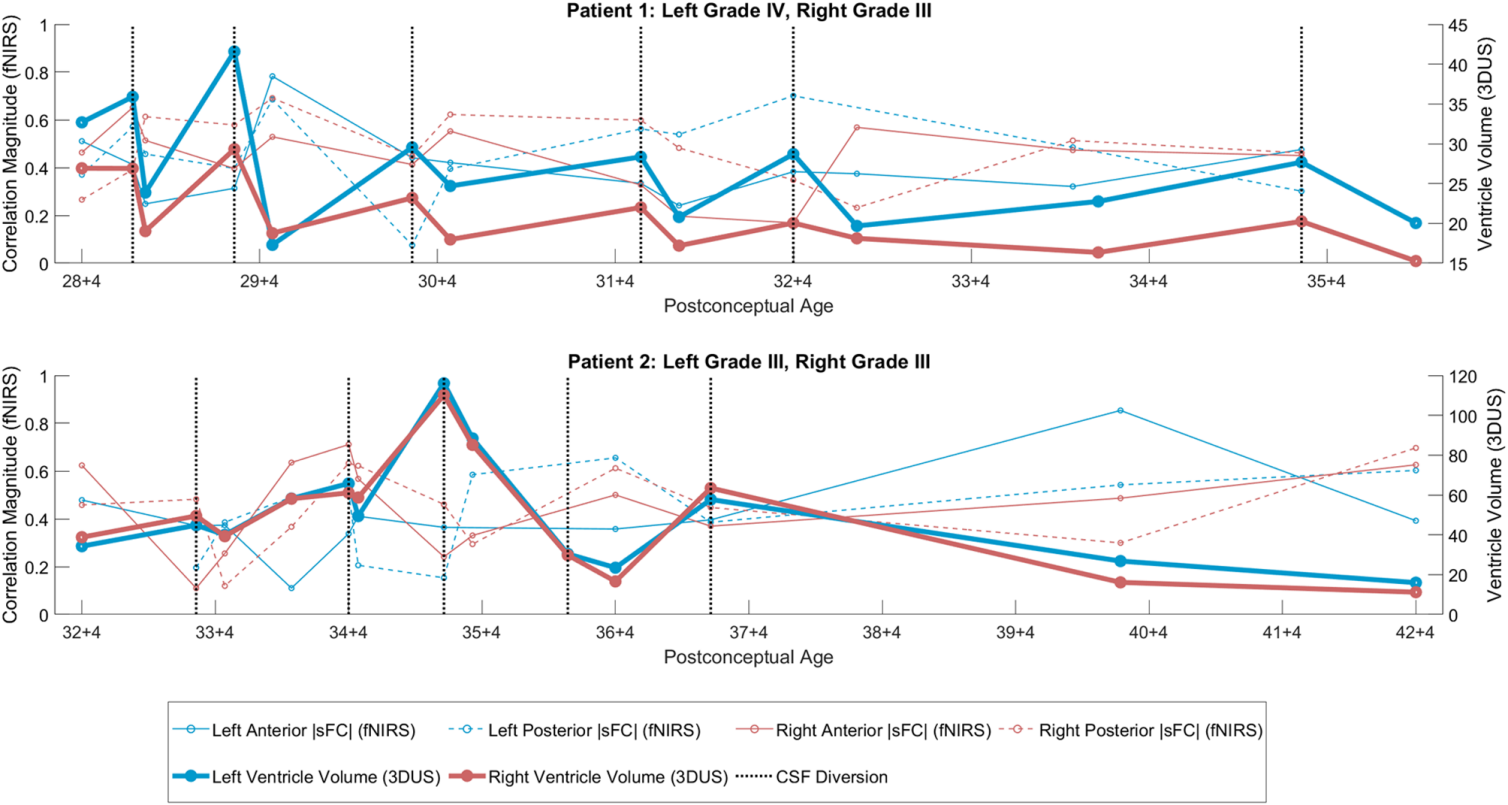
Two case studies from patients requiring CSF diversion. Vertical gray lines indicate CSF diversion.

To address our second aim concerning longitudinal assessments of 3D cUS VV in neonates with GMH-IVH and PHVD, we examined how they covary with sFC. Two of seven case studies from participants who underwent CSF diversion are presented in Fig. 7 (vertical grey lines indicate the timing of CSF diversion). An inverse relation between |sFC| and VV is evident even when CSF is diverted. Furthermore, increases in |sFC| following CSF diversions were often observed shortly after each procedure. For all neonates who underwent CSF diversion, left, right, and total VV decreased (Left: p < 0.001, mean = − 9.74, SD = 7.76; Right: p < 0.001, mean = − 10.20, SD = 8.98; Total: p < 0.001, mean = − 19.93, SD = 12.23). All clusters/regions, except the left posterior, had a mean increase in |sFC| but not significant at the group level (left anterior: p = 0.22, mean = 0.04, SD = 0.20; left posterior: p = 0.51, mean = − 0.00, SD = 0.27, right anterior: p = 0.10, mean = 0.07, SD = 0.19; right posterior: p = 0.21, mean = 0.06, SD = 0.27).

## Discussion

Despite obstetric and neonatal health improvements, GMH-IVH remains unacceptably high in preterm neonates. This study used a combination of bedside tools—3D cUS and fNIRS to monitor preterm infants with GMH-IVH from diagnosis until discharge. To our knowledge, no other study has incorporated these tools simultaneously. Our study aimed to determine if ventricular size in preterm infants with GMH-IVH was associated with changes in sFC. We observed an inverse relation between |sFC| and large VV: increases in VV were associated with decreases in |sFC|. Our findings of increased ventricle volume in preterm neonates and reduced fNIRS-based functional connectivity suggest that regional disruptions of ventricular size may impact the development of the underlying grey matter^53^. These results bear clinical significance given that the neonate's brain is rapidly growing and at risk for injury^54^.

In our first aim, in participants who did not have CSF diversion, the linear regression method produced a significant negative |sFC|/VV slope in the left hemisphere with a similar trend in the right, which indicates impaired sFC in preterm neonates when the ventricles were enlarged. We also observed similar but more compelling results in the participants who required CSF diversion: consistently negative across all three methods. The slopes in the "No CSF Diversion" group switched to positive under the LME-based methods, which suggests that most infants in this group had a positive relation (likely because most of these infants had mild GMH-IVH) and that the few negative outliers likely drove the initial linear regression result. Our findings suggest that increasing ventricular dilatation is associated with reduced |sFC| in preterm infants with GMH-IVH. To put this into context, the larger the ventricles, the more likely sFC will be impaired^53^. While GMH, especially when severe, is known to cause pathological and functional consequences based on the extent of white matter injury^55,56^, we did observe similar findings in our study, more so in the group with severe ventriculomegaly needing CSF diversion. In a recent study, Tortora et al. showed regional impairment of cortical and regional grey matter perfusion in preterm infants with mild GMH-IVH^57^.

As expected in our second aim, in the participants who required the CSF diversion, we observed elevated VV and reduced |sFC| right before the CSF diversion procedures. Conversely, after the diversion, VV decreased, and |sFC| often increased, although the results were not statistically significant at the group level, likely due to a small sample size. Observed increases in |sFC| were observed shortly after each CSF diversion procedure, possibly correlating to a decrease in ICP. Our findings are consistent with others. In their study of 9 preterm infants, Norooz et al. reported an increase in the mean regional cerebral oxygen saturation (rcSO_2_) value after decompression (42.6 ± 12.9% before vs 55 ± 12.2% after decompression)^58^. Our findings are also comparable to Kochan et al. Their study, which included 20 VLBW premature infants with GMH-IVH, demonstrated that ventricular dilatation was associated with lower cSO_2_, suggesting a decrease in cerebral perfusion^59^. Other studies in preterm infants with PHVD requiring CSF diversion procedures have consistently reported similar findings^60,61^. Similarly, Rajaram et al. and McLachlan et al. showed that cerebral blood flow increased after ventricular taps, further reinforcing our sFC trends^62,63^.

Our findings show that fNIRS may provide additional clinical information, particularly in preterm infants with PHVD, to help determine the optimal time for CSF diversion. While the definitive treatment of PHVD is VP shunt placement to divert CSF, this procedure is often delayed. During this delay, temporizing procedures are done to decrease ICP. However, a prolonged increase in ICP can lead to brain damage that can impact both periventricular WM and cerebral grey matter. Furthermore, the timings of the temporizing CSF diversion procedures mentioned above are unpredictable and rely on subjective clinical signs, symptoms and 2D cUS measurements. fNIRS measurements, therefore, have the potential to aid in timely decision-making.

Our study has some strengths that are worth highlighting. A key strength of this study was the serial measurements. To date, 2D cUS remains the neuroimaging modality of choice for bedside GMH-IVH diagnosis. Current guidelines recommend the first 2D cUS by the first week of life to screen for GMH-IVH. After that, repeat cUS is completed at 4–6 weeks of life to screen for complications, mainly periventricular leukomalacia. Recent evidence recommends more frequent screening, particularly amongst very preterm infants, to help identify these complications and for timely intervention for those needing it^64^. Moreover, timely diagnosis of these complications provides a window of opportunity to have meaningful conversations with parents regarding prognosis and plan for early intervention for the affected infants^65^. Another strength of our study was the demonstration of functional connectivity in preterm neonates using multichannel fNIRS, enabling us to cover multiple brain areas simultaneously. Lastly, our NICU and Paediatric Neurosurgery team have incorporated 3D cUS for monitoring preterm infants with GMH-IVH^40,41,44,66^ for the past decade. Our current 3D cUS is semi-automated, requiring time to segment the lateral ventricles. For 3D cUS to play a role in diagnosing and monitoring PHVD in other clinical settings, an efficient and fully automated segmentation algorithm is crucial. Several other groups have adapted this technique, given its reliability in VV estimation^67,68^. Nevertheless, we still achieved reliable VV estimates using our current system.

Some limitations of this study need to be acknowledged. First, the variability in the number of study measurements each participant had led to variability in the results. Our analysis showed a more robust and reliable sFC pattern when the study participants had three or more sessions. More sessions for each patient and fewer missing/excluded data would have enabled us to use more robust connectivity methods such as graph theory. Despite this, we still observed meaningful sFC patterns. Second, given the limited period, including follow-up data from this cohort was not possible. Morbidity is substantial in preterm infants with severe GMH-IVH and PHVD. Grade III and PHVI have been linked to CP, neurosensory problems and cognitive impairments; hence, it is only fitting that these outcomes are reported. Participants from this study are currently undergoing follow-up in the developmental follow-up and Paediatric Neurosurgery clinics. The neonates’ developmental outcomes will provide additional information regarding the clinical application of these tools and their implications. Third, the right hemisphere was inconsistent across linear regression in the "No CSF Diversion" group. This inconsistent finding could be attributed to a poor signal in one of the sources during the initial data acquisition period that may have influenced the results in the right hemisphere. Another plausible reason could be heterogeneity in our study participants (63.3% had mild GMH-IVH). Future prospective studies should examine larger populations of infants with all grades of GMH-IVH. Lastly, some neonates received antenatal steroids, and we don't know if this exposure influenced the results because we had a small sample. This, too, could be an area for future investigation.

We included a model with gestational age because it is reasonable to expect an increase in both VV and |sFC| across GA. We wanted to investigate their relationship that is separate from this trend. However, we did not observe any reliable main effects of GA, even in the milder “No CSF Diversion” group. It is likely that these effects were present but could not be detected due to a combination of low sample size and the noise introduced by IVH-related fluctuations in VV and |sFC|. In addition, these findings are not surprising given that with mild GMH-IVH, we expect to see the resolution of the bleeds with time and hence restoration of sFC. Previous studies have shown the maturation of sFC with advancing GA^69^. Further studies are required to determine if GA should be included in the model.

Other things to consider are poor spatial resolution and limited penetration depth (especially in preterm infants with dependent scalp edema). Such issues, however, are common in fNIRS studies, particularly in the very preterm population where the fit of the caps may be insufficient to make proper contact with the scalp. Therefore, future studies with larger cohorts are needed to confirm our results.

In conclusion, our study shows that 3D cUS and fNIRS are promising bedside tools for monitoring GMH-IVH and PHVD in preterm infants, providing complementary information about infants’ brain structure and function. Future research should explore the potential role of lateral ventricular volume size and spontaneous functional connectivity in the diagnostic and therapeutic approach to PHVD.

## Supplementary Information

Supplementary Information.

## Data Availability

The data that support the findings of this study are available from the corresponding author upon reasonable request.
