# Supplementary material for: Three-dimensional cranial ultrasound and functional near-infrared spectroscopy for bedside monitoring of intraventricular hemorrhage in preterm neonates
Source: Sci Rep. 2023 Mar 6;13:3730. doi: 10.1038/s41598-023-30743-4 (PMC9988970; doi:10.1038/s41598-023-30743-4)
Supplement: Supplementary file 1 — Supplementary Information. [file 41598_2023_30743_MOESM1_ESM.pdf]

Supplementary figures

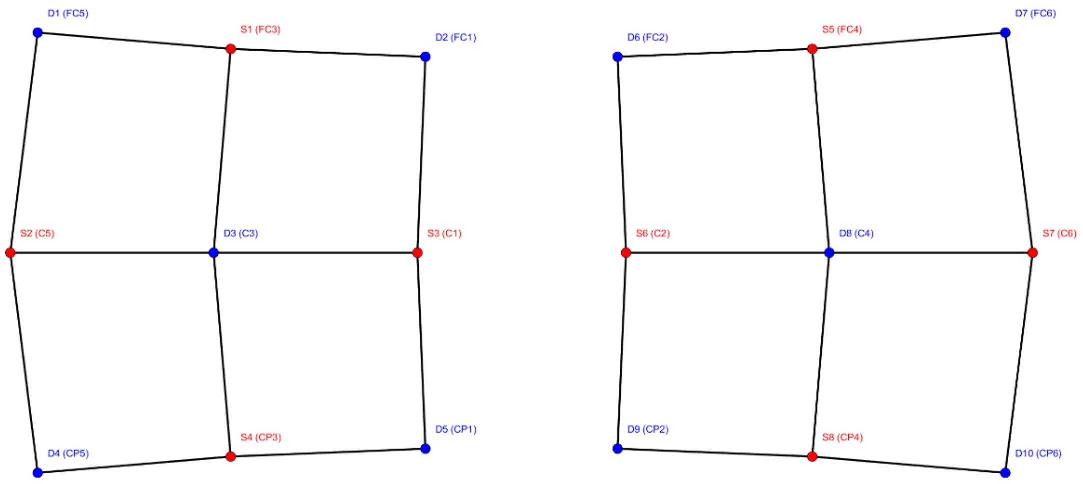

Supplementary Figure 1: *Montage*

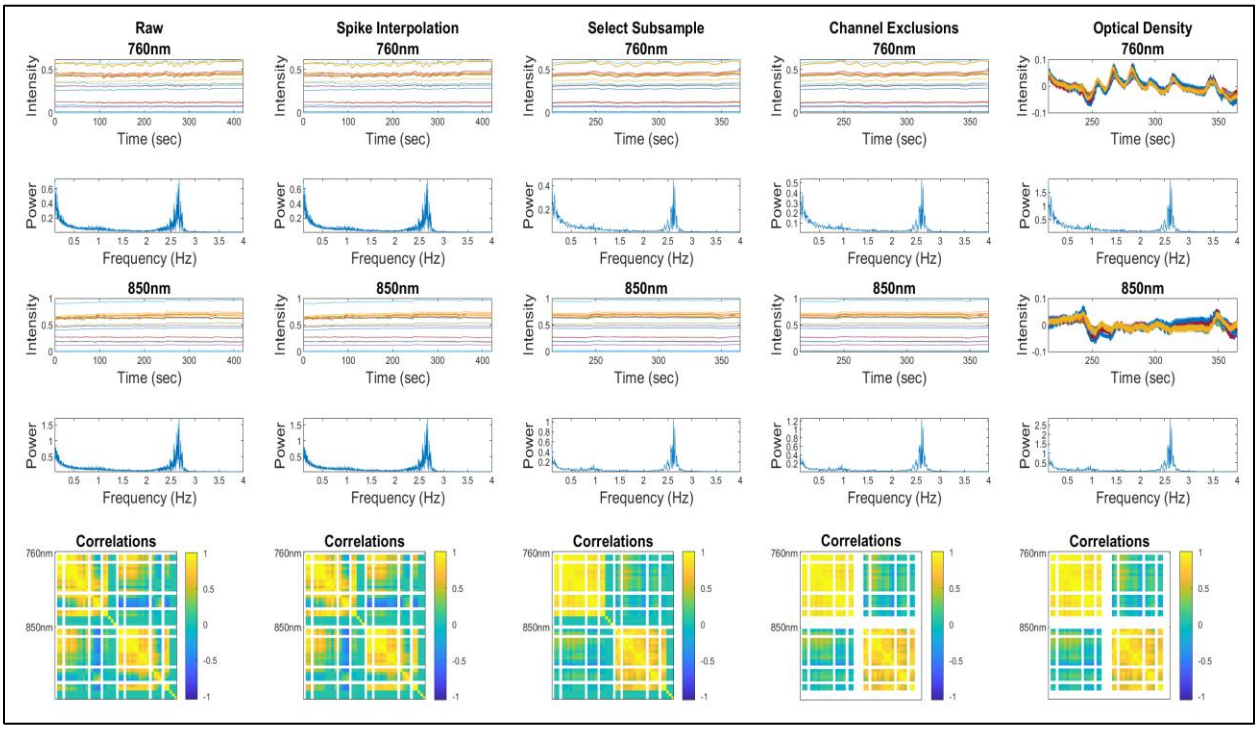

Supplementary Figure 2: *Preprocessing pipeline, from the acquisition of raw data to optical density*

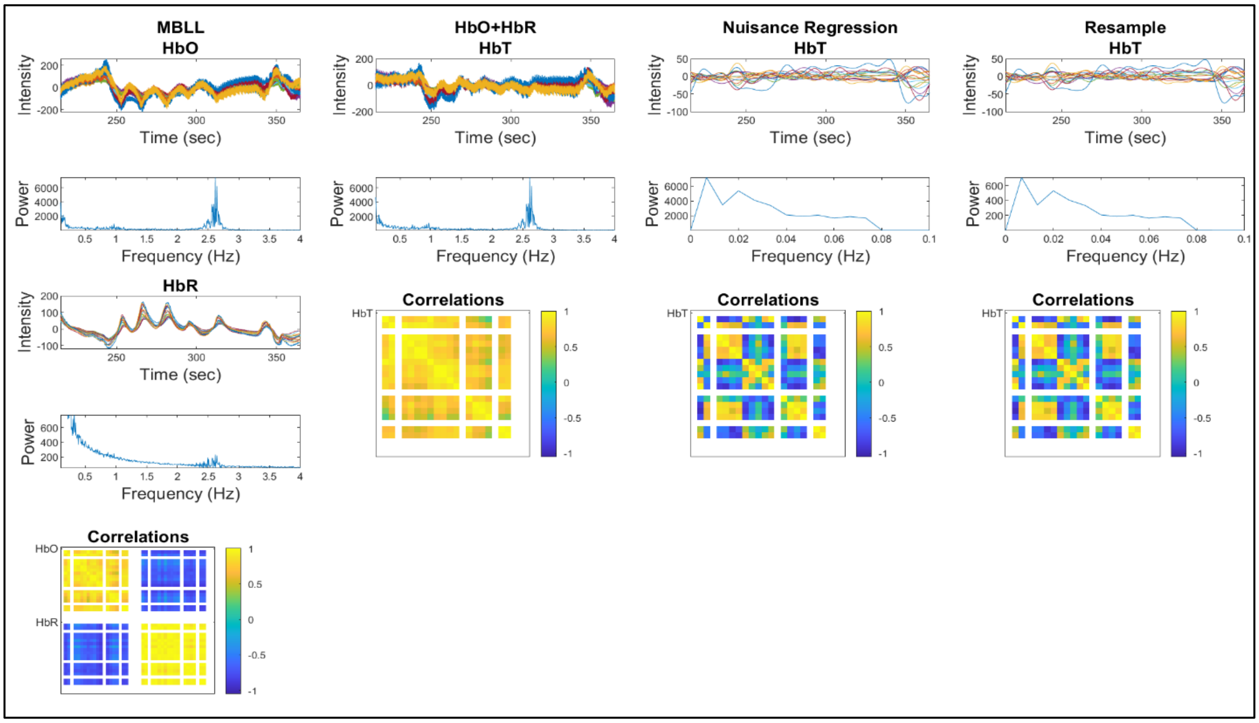

***Supplementary Figure 3**: Preprocessing pipeline (continued)*

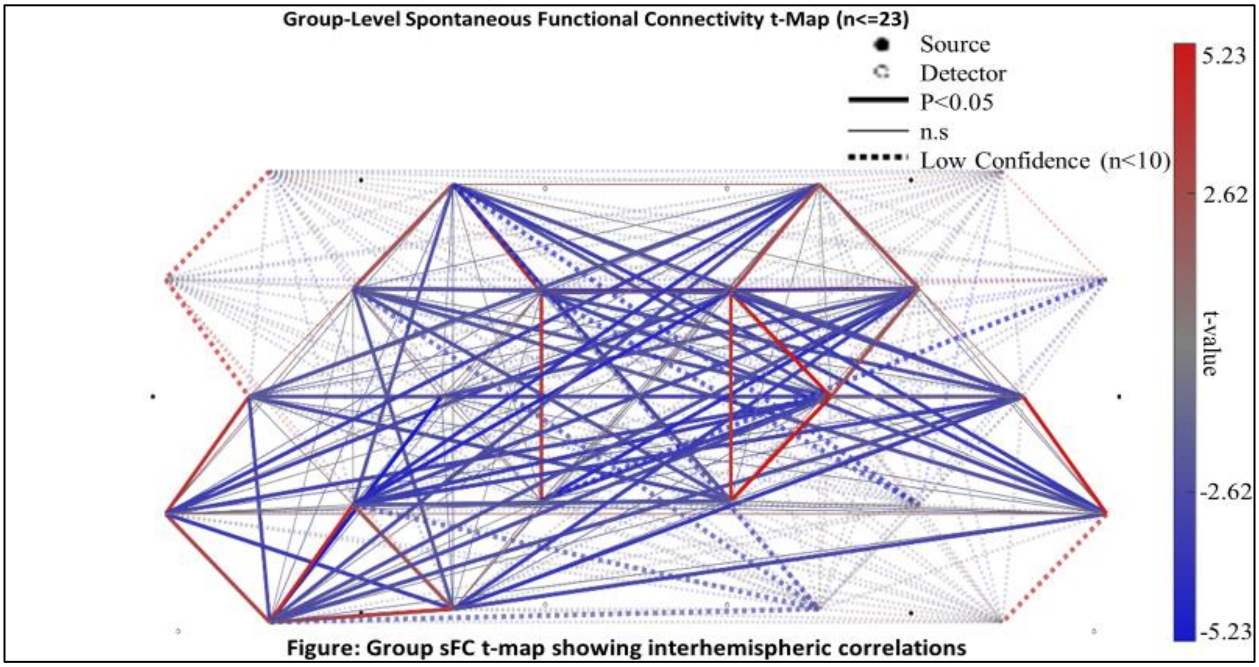

***Supplementary Figure 4:** Group t-map showing interhemispheric correlations*

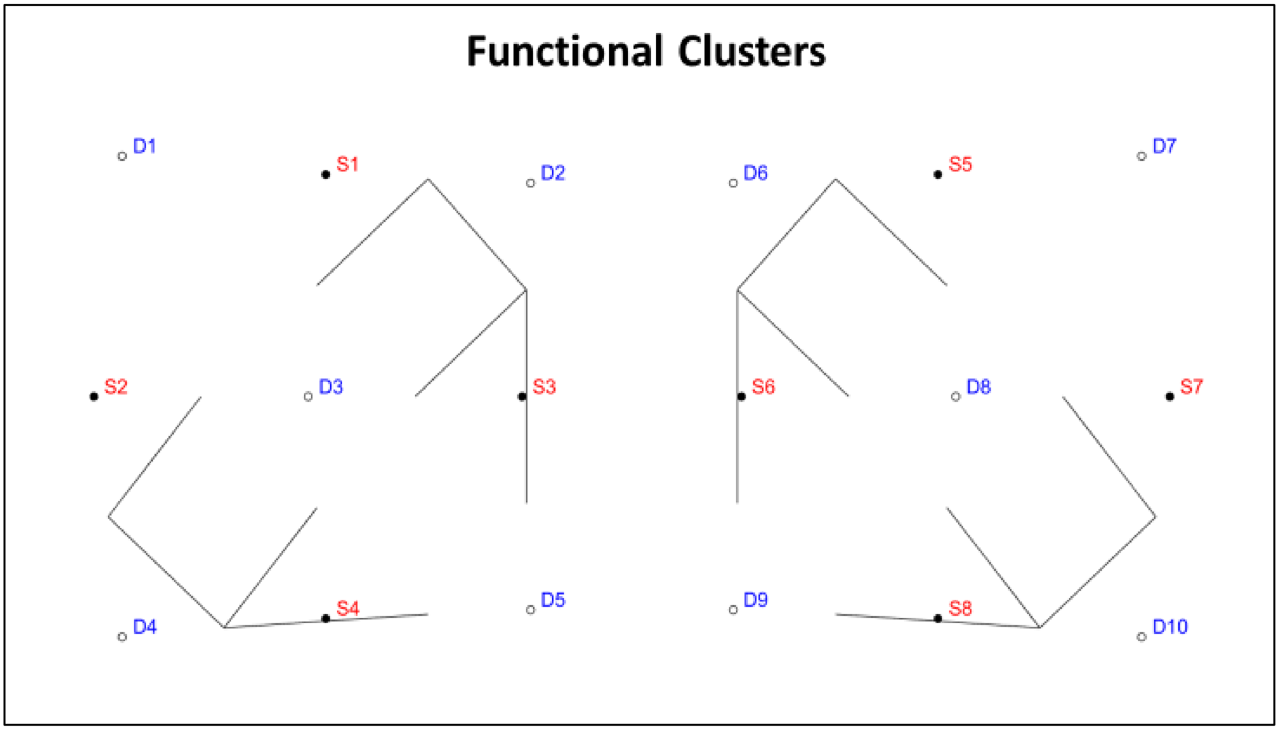

Supplementary Figure 5: *Four functional clusters*

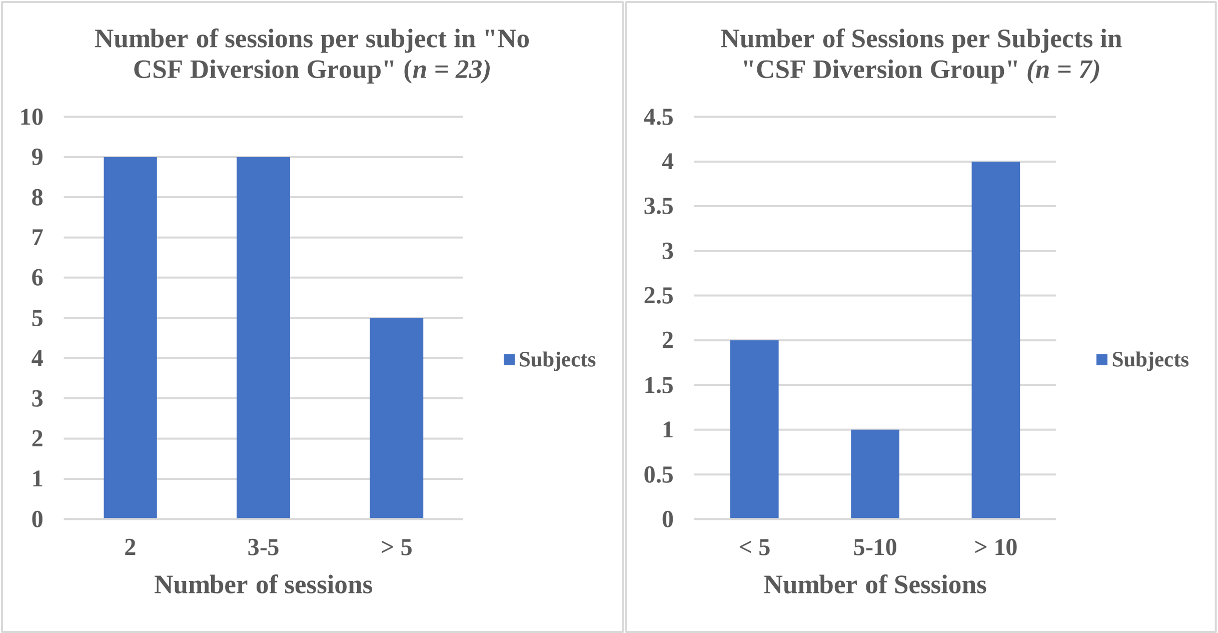

Supplementary Figure 6: *Figure showing number of sessions per patient in both groups*

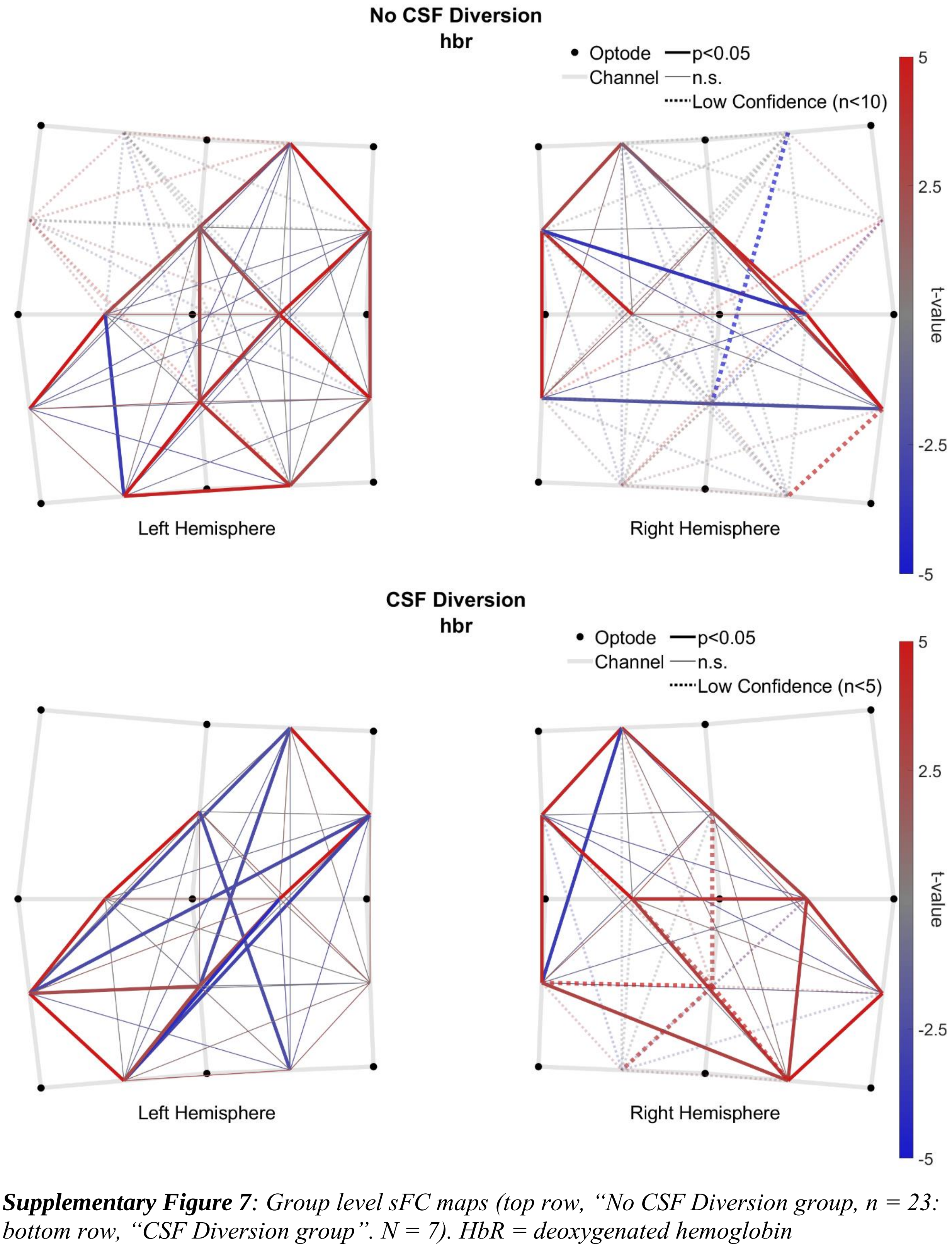

Supplementary Figure 7*: Group level sFC maps (top row, "No CSF Diversion group, n = 23: bottom row, "CSF Diversion group". N = 7). HbR = deoxygenated hemoglobin*

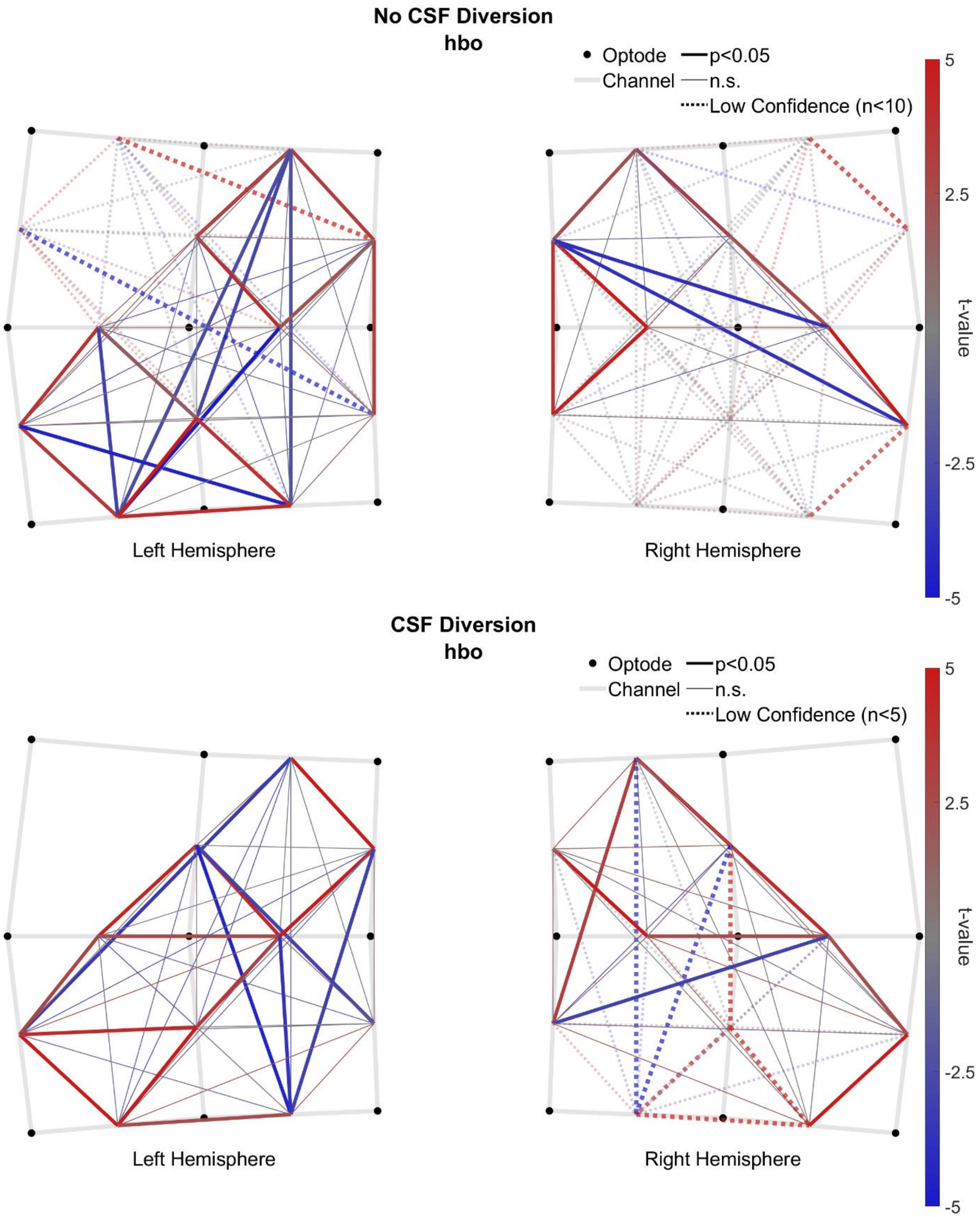

Supplementary Figure 8: *Group level sFC maps (top row, "No CSF Diversion group, n = 23: bottom row, "CSF Diversion group". N = 7). HbO = oxygenated hemoglobin*

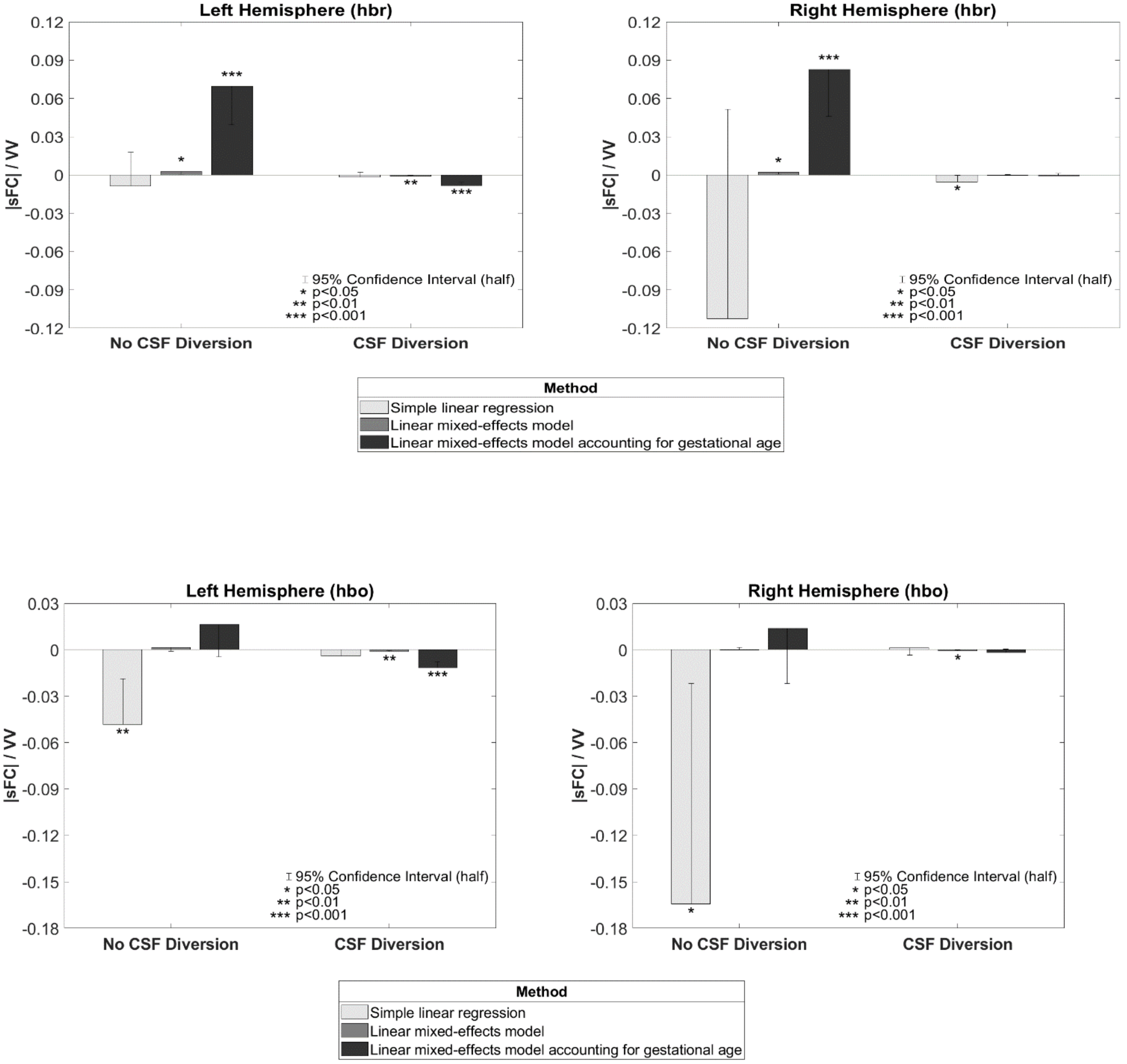

Supplementary Figure 9: *Hemispheric trends in group level sFC/VV from each slope method. Top row = deoxygenated hemoglobin (HbR). Bottom row = oxygenated hemoglobin (HbO)*
